# Supplementary material for: Interaction between the transmembrane domains of neurotrophin receptors p75 and TrkA mediates their reciprocal activation
Source: J Biol Chem. 2021 Jul 1;297(2):100926. doi: 10.1016/j.jbc.2021.100926 (PMC8327350; doi:10.1016/j.jbc.2021.100926)
Supplement: Supplemental Figures S1–S3 and Tables S1–S2 [file mmc1.pdf]

Supplementary Figures

Figure S1. Heterodimer formation Rotational correlation times t_c, measured for the individual amide cross-peaks of p75-TM-C257A, corresponding to the monomeric (blue bars), homodimeric (orange bars) and heteromeric (gray bars) states. At right, the mean t_c values for the three states are shown. t_c ranges, predicted for 1, 2 and 3 TM helices in DPC micelles based on the rotational diffusion data published for 14 TM domains (37) are shown by blue, orange and red rectangles, respectively.

Figure S2. Electron density of the POPC bilayer with TrkA and p75 TMDs. The density corresponding to water (blue), lipid headgroups (green) and acyl groups (black) are shown respect to the bilayer axis z-coordinate (in nm).

Figure S3. Molecular dynamics of the p75-AGA/III/TrkA TMD heterodimer.
A) Schematic representation of the spatial structure of the heterodimer p75-TMD-AGA/III (blue) and TrkA-TMD (orange) after 100 ns full-atom MD. The residues participating in the dimer interface are shown by blue (p75) and red (TrkA). B) Distance between TrkA-TMD and p75-TMD-AGA/III during CG-MD simulation time.

Figure S1

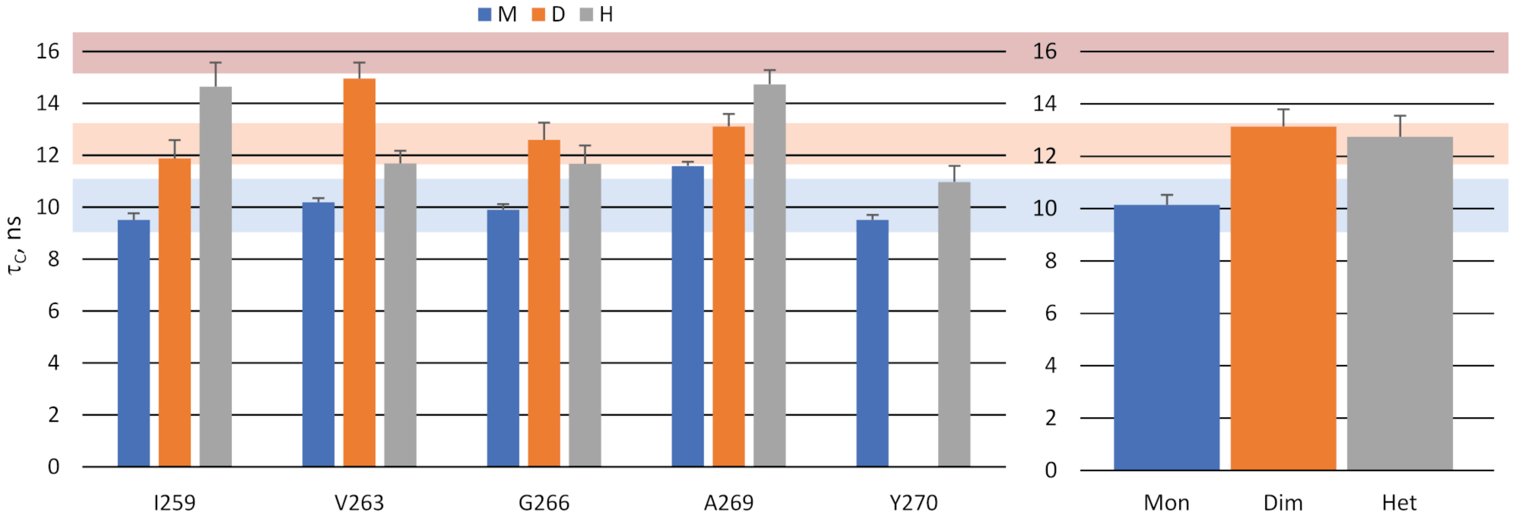

Figure S2

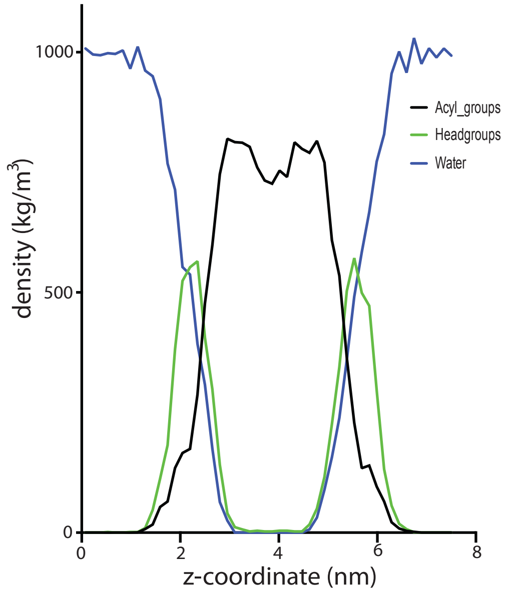

Figure S3

A

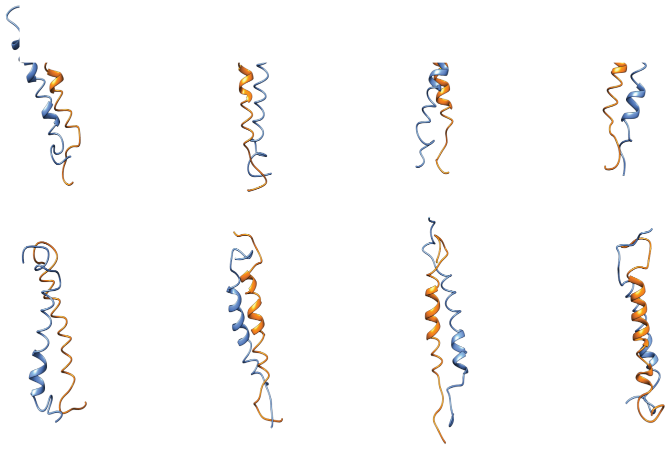

B

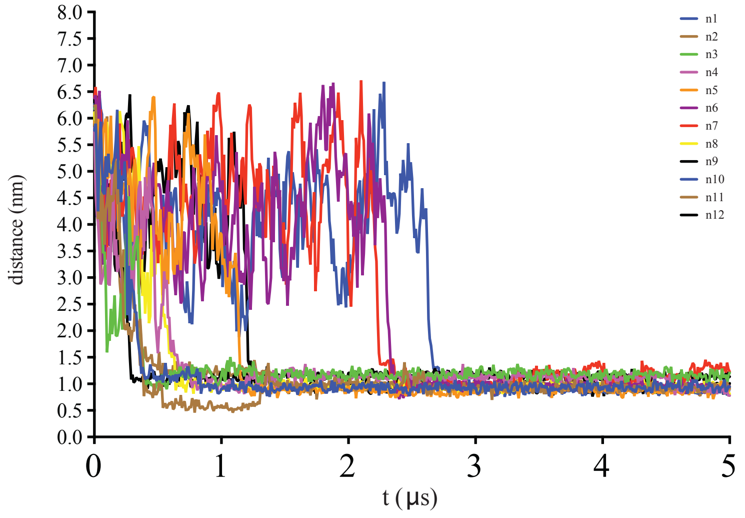

Table S1. Summary of performed simulations for p75 TrkA TMDs.

Model	Total atoms	POPC molec.	Water molec.	Simulation time	Number simulations
P75/TrkA CG-MD	8484	317	4100	10 μs	12
P75-AGA/TrkA CG-MD	7519	274	3712	10 μs	12
P75/TrkA FA-MD	93044	317	16400	100 ns	3

Table S2. Area per lipid (APL) and thickness of the lipid bilayer of the CG-MD simulations. See Material and Methods.

Simulation(CG-MD)	APL (Å^2)	Thickness (Å)
1	63.6	34.8
2	62.6	34.8
3	63.9	34.6
4	63.5	34.5
5	62.6	34.9
6	63.4	34.6
7	64.6	34.6
8	62.8	35.0
9	62.9	35.0
10	63.3	34.8
11	63.8	34.7
12	62.4	35.1
Average	63.2	34.8
